# Does bride price harm women? Using ethnography to think about causality

**DOI:** 10.1017/ehs.2024.21

**Published:** 2024-05-03

**Authors:** Eva Brandl, Heidi Colleran

**Affiliations:** 1 Lise Meitner Research Group BirthRites, Max Planck Institute for Evolutionary Anthropology, Leipzig, Germany; 2 Department of Human Behavior, Ecology and Culture, Max Planck Institute for Evolutionary Anthropology, Leipzig, Germany

**Keywords:** marriage, bride price, gender, ethnography, Melanesia

## Abstract

Many institutions claim that bride price – where the groom's family transfers wealth to the bride's family at marriage – harms women. Owing to its long-term engagement with communities that practise bride price, ethnography is well placed to identify causal mechanisms at play in this issue, and there is a substantial literature on its effects in a variety of world regions, including Melanesia. Here, we condense this literature, drawing out key causal arguments made about bride price in various Melanesian societies. This reveals a complex, multi-causal picture: rather than being singularly harmful, bride price may involve a mixture of drawbacks and benefits, making it a double-edged sword with contested implications. Bride price may constrain women's options before and during the marriage but also serves as a safety net that enhances their status. Its effects are probably influenced by many other variables, including age, kinship networks and residence structures. These dynamics have been transformed by conversion to Christianity, the (post-)colonial state, market integration, urbanisation and formal education, often yielding ambiguous outcomes. Rather than reducing ethnography to a collection of datapoints, we show that it can serve as a source of verbal arguments that can be used to challenge reductive narratives about sensitive issues and to formulate hypotheses for testing with quantitative data.

## Graphical abstract

**Figure:**
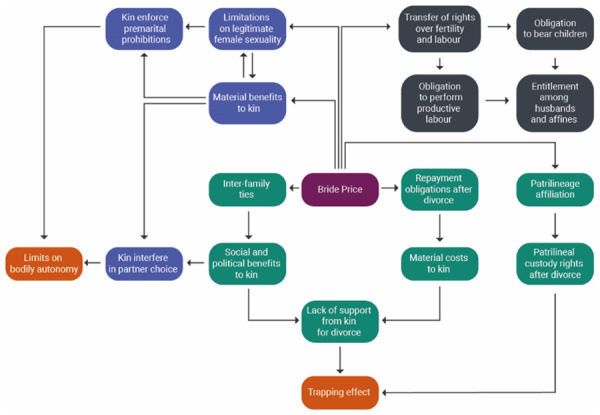

**Social media summary:** Bride price is often said to harm women. Here, we use ethnography to refine our understanding of causality.

## Introduction

Bride price, or the practice of transferring wealth from the groom's to the bride's family upon marriage, is widespread in Melanesia, where most marriages involve transfers of ‘traditional’ (e.g. pigs or mats) and non-traditional items (e.g. money) (see Box 1). Bride price has come under attack from international bodies and non-governmental organisations such as the United Nations, the European Union, the Secretariat of the Pacific Community, Human Rights Watch and Oxfam, which portray it as a ‘harmful cultural practice’ that reinforces gender inequality and gender-based violence (see Box 1). Similarly, international news media have described bride price as a form of slavery that subjugates women to their husbands (Galbraith, 2011). Indeed, many Melanesian countries experience high levels of gender-based violence (Salomon & Hamelin, 2008; UN HRC, 2013; UN CEDAW, 2016; Homan et al., 2019): 60% of ever-partnered women in Vanuatu have experienced intimate partner violence while in Bougainville, Papua New Guinea, 80% of ever-partnered men are perpetrators (VWC, 2011; Fulu et al., 2013). Accordingly, the United Nations and Human Rights Watch have demanded that bride price be banned or made unenforceable (Barr, 2015; UN CEDAW, 2016).

Bride price has also become somewhat controversial within many Melanesian societies, where there is lively debate about the relationship between local *kastom* (traditional life-ways) and institutions introduced under colonialism, such as Christianity, the state and the market economy. At the same time, *kastom* is not an unchanging pre-colonial tradition, but a dynamic set of political agendas that combine a post-colonial enthusiasm for preserving tradition with a syncretistic tendency to incorporate foreign ideas such as Christianity into local ideologies. Debates about *kastom* are increasingly influenced by international women's rights activism, raising concerns about the role of women in society.

**Figure 1.**
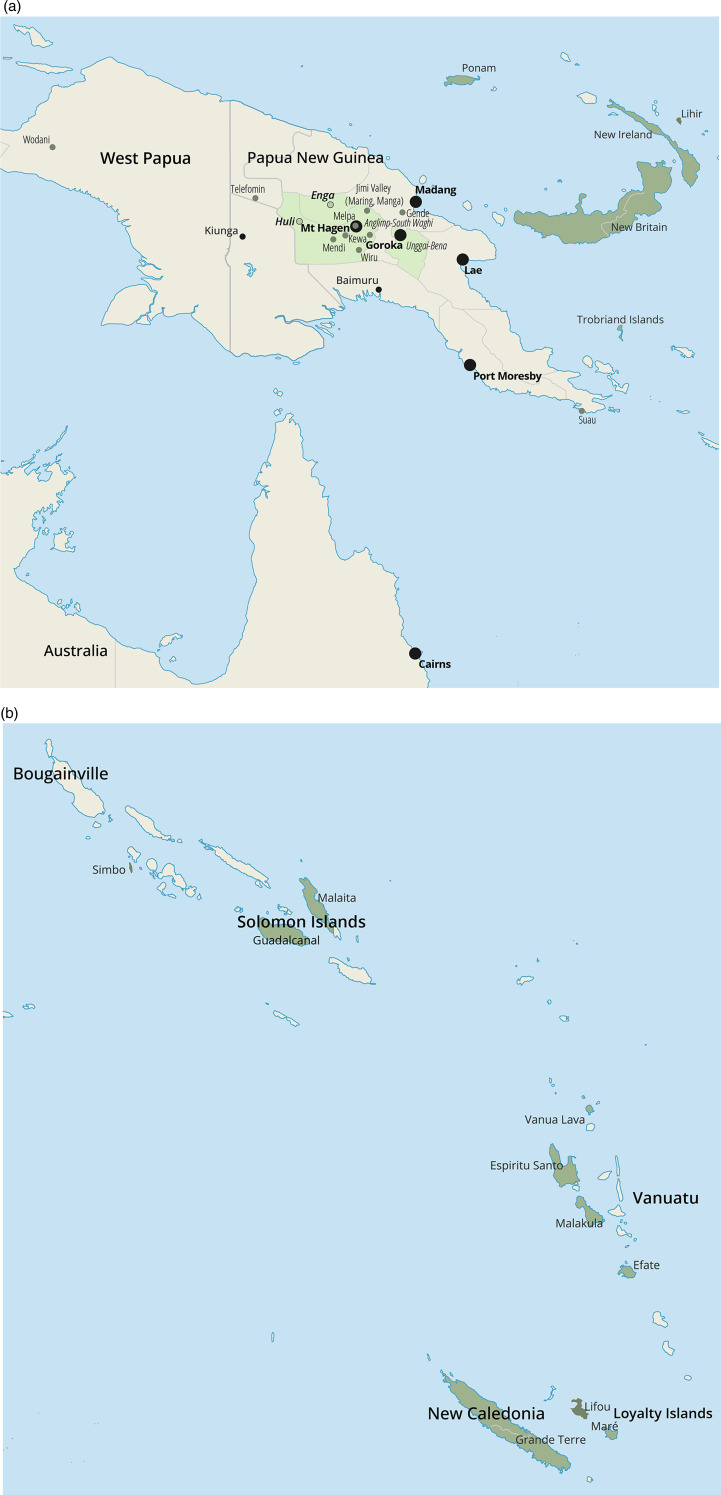
(a) Field sites in Australia, Papua New Guinea and West Papua covered in this review. Dark green colouring stands for islands and light green for mainland districts. Large black dots mark mainland cities, small black dots mark smaller settlements and green dots mark cultural groups. Manus is not covered in our review but coloured on the map to indicate the location of Ponam. (b) Field sites in the Solomon Islands, Vanuatu and New Caledonia covered in this review. For a detailed breakdown of the different field sites and the publications that cover them see Supplementary Table S1, SI.

The claim that bride price causes harm is therefore culturally and politically sensitive. Yet is there evidence for it? Research on other ‘harmful practices’ such as early marriage or polygyny suggests that we should be cautious: they are not always associated with negative outcomes, and even if they are, this often comes down to confounding by other variables (Lawson et al., 2015; Baraka et al., 2022). Similarly, quantitative studies on bride price have produced mixed and sometimes contradictory findings (see Box 2, Supporting Information, SI). We therefore turn to ethnography to improve our understanding of the impact of bride price on women. Anthropologists have produced a rich ethnographic record about bride price in many different Melanesian cultures, along with a long history of theorising how it affects women's status. Often, when researchers outside socio-cultural anthropology engage with the ethnographic record, it is treated as a source of datapoints: qualitative information is hard-coded into categories (such as 1 = practising and 0 = not practising bride price) to test hypotheses about cross-cultural trends developed in other fields. Yet ethnography is not just a collection of data. It is also a treasure trove of *arguments*. While the term ‘causality’ is rarely used, these verbal arguments nonetheless advance explanations of cultural phenomena. These informal models can serve as a resource for other fields: they can help us think critically about causality, build better-informed models of the associated dynamics and enhance study designs by generating hypotheses that can be tested with quantitative data. Owing to its intensive, long-term engagement with communities, ethnography is especially well placed to identify the mechanisms at play in sensitive domains. We therefore take ethnographic claims about bride price for what they are: social theories about how it affects women's status in the family and in society, the forces that shape these dynamics and how they have changed over time. Accordingly, we do not aim to construct a new master-narrative about bride price, but to distil core causal claims made in a wide selection of ethnographic works, highlighting points of consensus and disagreement, identifying variables and predictions for further testing, and presenting them in a systematic manner, so that researchers from other fields can draw on them. In what follows we identify causal arguments about bride price in various Melanesian countries, including Papua New Guinea, the Solomon Islands, Vanuatu, New Caledonia and West Papua, and among Melanesian expatriates in Australia (see Figure 1a and b). To capture a broad range of places, we conducted a literature search based on a snowball sampling strategy: (a) starting with papers that appeared in a special issue on bride price and women's autonomy published in *Oceania* (Sykes & Jourdan, 2020), we then (b) reviewed references cited in those papers based on word searches where possible, prioritising publications mentioning ‘bride price’, ‘bride wealth’ and alternative spellings like ‘bride-price’ (for French publications we used ‘prix de la fiancée’, ‘prix de la mariée’ and ‘dot’). Finally, we (c) reviewed additional publications encountered during the literature search. We prioritised recent publications (from the last 50-odd years) over classic monographs to keep up with current events.

Our review is divided into four parts. First, we present claims about the drawbacks and benefits of bride price for women. We then address factors that influence how bride price shapes women's status. Third, we review how social changes associated with ‘development’ reshape the relationship between bride price and women's status. In our conclusion, we illustrate how we can use these arguments to identify variables, generate hypotheses and build causal models that can be tested with quantitative data.

Box 1. Terms and definitions

**Bride price:** also known as ‘bridewealth’, a marriage payment where the family of the groom transfers goods to the family of the bride (Goody & Tambiah, 1973; see also Tambiah, 1989; Mulder, 1989, 1995). It formalises the relationship between husband and wife, demonstrates their families’ approval for the marriage and builds ties between the two families (Dalton, 1966). In many cases, the husband's side also acquires rights over the wife's labour and fertility; additionally, bride price compensates the wife's family for losing her and formalises the legal status of the couple's children (Dalton, 1966). In many Melanesian societies, the bride's family can waive the bride price and a customary marriage can therefore be valid without it (Luluaki, 1997).

**Melanesia:** geographical region in the South Pacific comprising Papua New Guinea, Vanuatu, New Caledonia, the Solomon Islands, West Papua and Fiji. Although it is a highly diverse region that has been subject to dramatic social change, and although the term itself is of colonial origin, local scholars and residents now use the term ‘Melanesia’ to describe what has come to be seen as a legitimate sub-regional identity within the Pacific (Lawson, 2013).

**Harmful cultural practice:** defined by the United Nations as ‘discriminatory practices committed regularly over such long periods of time that societies begin to consider them acceptable’ (UNICEF, 2023). The United Nations, the European Union, the Secretariat of the Pacific Community, Oxfam and Human Rights Watch have argued that bride price constitutes such a practice because it reinforces gender inequality and gender-based violence (SPC, 2009; VWC, 2011; UN HRC, 2013; Barr, 2015; UN CEDAW, 2016; DEV EU, 2019; Homan et al., 2019). According to these institutions, putting a ‘price’ on a woman commodifies her, violates her human rights and treats her like property (UN CEDAW, 2005; Barr, 2015; HLPF, 2020). For example, Human Rights Watch states that bride price has negative consequences because men believe it grants them ownership over their wives (Barr, 2015). Human Rights Watch therefore includes it alongside polygamy and witchcraft accusations in a list of ‘harmful, discriminatory practices that both contribute to family violence and impede survivors from seeking help’ (Barr, 2015: 53). The UN states that the root cause of gender-based violence is found in ideologies that legitimate male violence towards women (UN HRC, 2013). It argues that bride price, alongside other causes such as polygamy, presents ‘an aggravating factor that fuels situations of domestic violence’ (UN HRC, 2013: 6) by encouraging men to view women as property (UN HRC, 2013). Finally, Oxfam states that the root cause of gender-based violence is gender inequality (Homan et al., 2019). They argue that bride price enables inequality by reinforcing notions of male ownership over women (although they concede that it may have been more about building ties in the past) (Homan et al., 2019).

## 1. The double-edged sword of bride price

### 1.a. Drawbacks for women

Bride price legitimates female sexuality under the rules of alliance that govern social groups. Marriage is therefore not just about the relationship between husband and wife, but about the interests of a wider network of kin on both sides. Women are expected to employ their reproductive capacities to support marriage, kinship,and social reproduction within this system; relationships that could undermine social reproduction are stigmatised (Salomon, 2000; Wardlow, 2002, 2006a, b). Bride price may therefore restrict women's autonomy before marriage, encouraging a girl's relatives to monitor her premarital conduct and to exercise pressure on partner choice. Girls were often (and sometimes still are) expected to guard their modesty until marriage, in part because amounts were higher for virgins: premarital transgressions could spoil a girl's marriage prospects (Burt, 1988; Jourdan & Labbé, 2020). A girl's kin can also prevent her from formalising a relationship by asking for exorbitant payments (Jourdan & Labbé, 2020). Because marriages build ongoing ties between families, they are socially and politically important for the couple's relatives, and the chance to obtain wealth also creates economic incentives for the bride's family (Wilson, 1987; Luluaki, 1997; Henry & Vávrová, 2016). As a result, a girl's relatives may pressure her to marry a particular man, a concern echoed by local scholars and residents (Wilson, 1987; Luluaki, 1997; Filer, 1985).

Bride price may also constrain women in two important ways during the marriage. First, ethnographers have argued that it can embolden the husband and his relatives to believe they have a right to control the wife's labour and fertility (we call this ‘entitlement dynamics’). Second, ties between families, repayment obligations and associated custody rights may prevent women from leaving troubled marriages (we call this the ‘brideprice trap’).

First, bride price establishes a husband's rights over his wife's labour, sexuality and fertility, and, in patrilineal societies, incorporates her and her children into his kin group; this entails that the woman lives and cooperates with his family while the children are members of his lineage (Strathern & Strathern, 1969; Feil, 1981; Strathern, 1984; Pflanz-Cook, 1993; Goddard, 2010; Servy, 2020). Paying bride price is therefore sometimes interpreted as conveying ownership of a wife's productive and reproductive capacities, and the husband's kin may expect to exert power in the relationship once they have ‘paid’, especially if the bride price was high (Wilson, 1987; Rosi & Zimmer-Tamakoshi, 1993; Salomon, 2000; Macintyre, 2011; Lepani, 2016). The wife is expected to remain faithful, bear children, grow crops and cultivate ties between the two families; as a result, her husband and in-laws may make demands and feel entitled to control, disrespect and abuse her, a concern shared by local scholars and residents (Filer, 1985; Wilson, 1987; Burt, 1988; Salomon, 2000; Macintyre, 2011; Gibbs, 2016; Biersack, 2016; Lepani, 2016; Jourdan & Labbé, 2020; Buchanan-Aruwafu et al., 2003; Buchanan-Aruwafu & Maebiru, 2008; Servy, 2020). For example, Manga men in Papua New Guinea could inflict violence on adulterous wives once they had paid bride price, at least in the past (Pflanz-Cook, 1993). Others argue that, rather than being an inherent component of bride price, these entitlement dynamics are the result of replacing traditional gifts with cash (see Section 3.c). Either way, women's families use various counter-strategies to mitigate them, for example by giving return gifts (from the bride's to the groom's family, made after the bride price has been given and which are customary in some societies) to showcase their prestige and make the bride less indebted to her in-laws, or by no longer demanding bride price at all (Henry & Vávrová, 2020; Jourdan & Labbé, 2020).

Second, bride price can trap women in unhappy marriages. Customary divorce involves not only the couple but also their families (Brown & Care, 2005). The husband's family can request that the bride's kin repay the bride price, preventing women from leaving if their relatives are unwilling or unable to pay for the divorce and pressure them to return to their husbands (Panoff, 1978; Wilson, 1987; Rosi & Zimmer-Tamakoshi, 1993; Zimmer-Tamakoshi, 1993; Goddard, 2010; Jourdan & Labbé, 2020; Neuendorf, 2020; Henry & Vávrová, 2020). The woman's situation is particularly problematic when the bride price was high or she has not met expectations (Wilson, 1987; Rosi & Zimmer-Tamakoshi, 1993; Zimmer-Tamakoshi, 1993; Henry & Vávrová, 2020). Moreover, since bride price establishes ties between families that benefit the bride's relatives, they are reluctant to rupture these with divorce (Wilson, 1987; Salomon, 2000; Henry & Vávrová, 2020). Some ethnographers caution that bride price does not cause this directly but concede that it strengthens ties in ways that can trap women in troubled marriages (Henry & Vávrová, 2020), so there may be an important indirect relationship. Finally, in patrilineal societies, children are affiliated with the father's lineage, bride price establishes legal paternity and thus the father's rights over children, and his kin provide access to land and other heritable resources (Zorn, 2010a; Corrin, 2016; Jourdan & Labbé, 2020). The husband therefore typically retains custody after bride price is paid, further preventing women from leaving (Care & Brown, 2004; Corrin, 2016; Neuendorf, 2020; Henry & Vávrová, 2020; Jourdan & Labbé, 2020). Without bride price, the husband and his family have less leverage over the woman, making it easier for her to return to her relatives (Neuendorf, 2020).

Additionally, the ethnographic record suggests that bride price can be a major financial stressor for men, leading them to lash out against women. High demands from the woman's relatives can prevent men from formalising a relationship if they or their family are unable or unwilling to pay (Jourdan & Labbé, 2020). In many cases, grooms need help from their relatives to pay for the bride price; once married, they are indebted to their donors and must reciprocate, which is a financial burden (Jourdan & Labbé, 2020). Men who receive no help from their relatives are of course also burdened (Rosi & Zimmer-Tamakoshi, 1993). Bride price can therefore drain wealth from young men and breed resentment against elders, especially if the parents of a woman they like reject them for a wealthier rival (Filer, 1985). Among the Huli in Papua New Guinea, this even incentivises assault (Wardlow, 2006a). Men who have ‘stolen’ an unmarried girl with premarital intercourse must compensate her family for damaging her reputation and marriage prospects, ideally by marrying her (Wardlow, 2006a, b). Fearing that a love rival will assemble the bride price before they do, some men assault the woman they want to marry to secure the match (Wardlow, 2006a).

### 1.b Advantages for women

**Figure 2.**
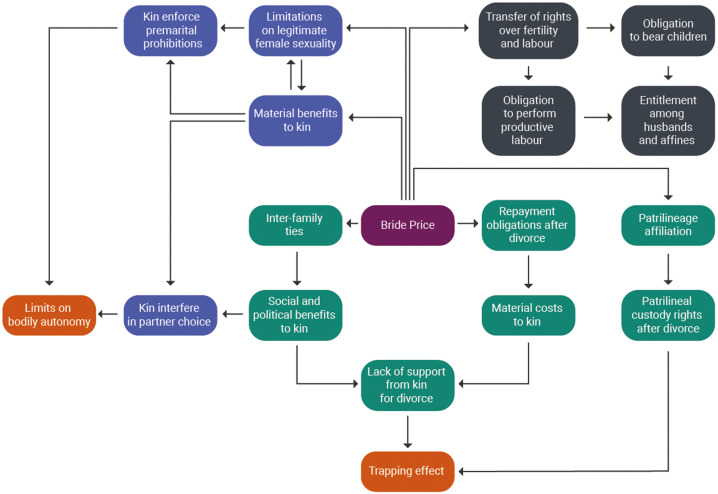
Schematic of drawbacks attributed to bride price. Bride price (in purple) is positioned in the centre; arrows point outwards to indicate different sets of arguments in the literature; mechanisms invoked in these arguments are clustered into particular causal pathways and social incentives (green = obstacles to divorce, black = marital pressures and obligations, lavender = premarital restrictions); consequences for women (in orange) are positioned at the end of each pathway. Drawbacks and benefits are also summarised in Supplementary Table S2, SI. Note that this is not intended to be a testable model, but a summary of concepts mentioned in the literature.

**Figure 3.**
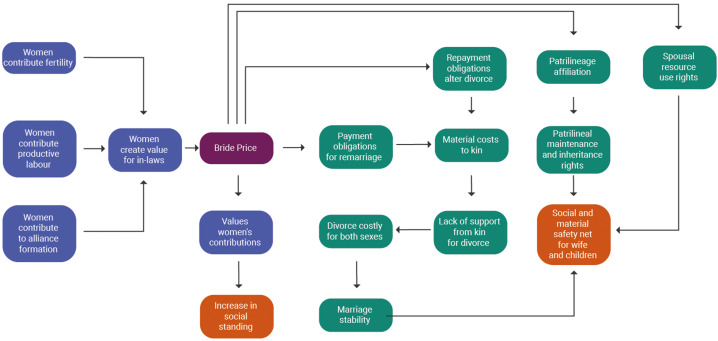
Schematic of benefits attributed to bride price. Bride price (in purple) is positioned in the centre; arrows point outwards to indicate different sets of arguments in the literature; mechanisms invoked in these arguments are clustered into particular causal pathways and social incentives (green = stability of investment by husbands and in-laws, lavender = social status and reputation); consequences for women (in orange) are positioned at the end of each pathway. Drawbacks and benefits are also summarised in Supplementary Table S2, SI. Note that this is not intended to be a testable model, but a summary of concepts mentioned in the literature.

While ethnographers have pointed out drawbacks and restrictions associated with bride price, they have also argued that bride price can protect and empower women in two important ways (see Figure 2 and Figure 3 for a summary of these arguments). First, brideprice values women's contributions and may therefore raise their standing in the husband's family. Second, bride price may serve as a safety net by securing women's and children's rights to maintenance, support and resources.

Traditionally, bride price compensates the bride's family for raising and now losing her to marriage, alleviating their sadness and honouring the subsistence labour and fertility she will bring to the husband's family, which allow them to continue their lineage (Strathern & Strathern, 1969; Feil, 1981; Clark, 1991; Marksbury, 1993; Wardlow, 2002; Buchanan-Aruwafu et al., 2003; Guo, 2020; Henry & Vávrová, 2020; Servy, 2020). Through marriage, the wife strengthens ties between families and assumes a central role in social reproduction, and so her status and agency increase once married (Paini, 2020). While researchers have argued that high bride prices can fuel abusive entitlement dynamics and trap women in troubled marriages (see Section 1.a), they have *also* argued that they improve the woman's status among her in-laws (Köngäs Maranda, 1974; Henry & Vávrová, 2020). Women with a low bride price may have a lower status, are disrespected in the husband's household and are looked down upon by other women who have married into the husband's group (Köngäs Maranda, 1974; Henry & Vávrová, 2020). Accordingly, it may not be true that high bride prices are always risky whereas low ones are always ‘safe’; instead, they may be associated with different kinds of risks.

Bride price may also create a safety net: it establishes legal paternity and secures women's and children's access to commitments from the husband's side. Bride price thereby ensures the wife's right to maintenance, along with lineage membership, inheritance and custody rights for the children (Zorn, 2010a; Corrin, 2016; Jourdan & Labbé, 2020). In patrilineal societies, bride price incorporates the woman and her children into the husband's group (Pflanz-Cook, 1993). This enables women to use (and children to inherit) the gardening land of the husband's family (Stewart & Strathern, 1998; Sykes, 2013, 2018; Jourdan & Labbé, 2020). Formalising relationships with bride price therefore clarifies the rights and obligations of both spouses (Goddard, 2010), which remains attractive. When unmarried Baimuru couples in Papua New Guinea fall pregnant, they often approach the woman's kin to formalise the union with bride price, which validates the woman's choices, puts the relationship on a more solid footing, secures material support from her partner and ensures he meets his obligations to her kin (Neuendorf, 2020). Moreover, bride price makes divorce costly for men if their relatives are unwilling to pay for a second marriage (VWC, 2011). Many local residents agree that bride price stabilises marriage, and that this benefits children (Filer, 1985). Women may also benefit from marriage stability if it secures them resources and social status and protects them from the social and economic risks associated with divorce. Nevertheless, marriage stability is arguably a double-edged sword in itself, and whether it really benefits women may differ depending on circumstances: while some women pursue bride price to solidify their relationship and secure support from a committed partner, others find themselves trapped with an abuser (see Section 1.a).

Either way, many women *want* to marry with bride price (Goddard, 2010; Neuendorf, 2020; Paini, 2020), and case studies suggest that breaking with established customs creates its own risks. In many areas of Papua New Guinea, the bride's kin have traditionally received bride price in a formal ceremony (Goddard, 2010). Low-income men in Port Moresby, who struggle to pay upfront, resort to more informal alternatives such as instalments or delayed payments, making the legal status of the relationship ambiguous (Goddard, 2010). This fuels disputes about payments and contributes to relationship breakdown (Goddard, 2010). Unions among professionals from different ethno-linguistic groups also deviate from *kastom*, for example by making non-traditional gifts or neglecting some in-laws (Sykes, 2018). These arrangements weaken ties between in-laws and can jeopardise access to land (Sykes, 2013, 2018). Women who opt out entirely may be even worse off. Some Huli women forego marriage in favour of promiscuous relationships and violate gendered modesty norms (Wardlow, 2002, 2006b). They are thereby thought to shame their relatives, disrupt social reproduction, abandon their kin obligations, and ‘eat their own bride price’ (using money they receive from boyfriends to buy commodities for themselves instead of bringing in bride price for their families) (Wardlow, 2002, 2006b). In many settings, women with a ‘licentious’ reputation lose the respect and protection of their kin and receive no support if they are victimised, which they are at high risk of (Wardlow, 2002, 2006b; Salomon, 2000; Salomon & Hamelin, 2008; Lepani, 2008; Kelly-Hanku et al., 2016). Moreover, ‘illegitimate’ children are sometimes left without a legal father or patriclan affiliation (Strathern & Strathern, 1969).

## 2. Impact of other factors

Bride price reflects complementary gender roles: traditionally, women bear children, grow crops and breed pigs while men provide gardening land and political leadership, and participate in ceremonial gift exchange (Strathern & Strathern, 1969; Strathern, 1984; Burt, 1988; Rosi & Zimmer-Tamakoshi, 1993; Zimmer-Tamakoshi, 1993; Salomon, 2000; Wardlow, 2002; Eves, 2019; Jourdan & Labbé, 2020; Wiessner & Pupu, 2021). Women rely on men to access land but men rely on women's labour to meet their exchange obligations and grow their prestige (Strathern, 1984; Burt, 1988; Zimmer-Tamakoshi, 1993; Salomon, 2000; Demian, 2017; Eves, 2019; Wiessner & Pupu, 2021). In some societies, men exchange valuables that do not depend on women's labour (Clark, 1991; Breton, 1999), but this appears to be rare. The husband and his family also depend on the wife's relatives to form exchange partnerships (Strathern & Strathern, 1969). In many Highland societies in Papua New Guinea, relationships with in-laws used to play an important role in large-scale gift-exchange systems that were politically significant (Merlan, 1988, although these have declined, see Stewart & Strathern, 2005). For Tombema-Enga, return gifts established a lifelong exchange partnership between the groom and the bride's family (Feil, 1981). Access to the social networks and material resources of the wife's relatives therefore increased the husband's social status (Feil, 1981). Through marriage, women forged such alliances between families, positioning them ‘in between’ their in-laws and natal kin and giving them an important role as intermediaries (Strathern & Strathern, 1969; Feil, 1981).

Men and women therefore need each other, but they are not necessarily treated equally. As in many other Highland societies, Kewa women produce valuables, but men appropriate these for gift exchange to increase their own prestige and exclude women from political leadership (Josephides, 1983, 1985). In such systems, bride price effects do not operate in a vacuum: other variables – such as women's age, parity and position in their social network, as well as kinship systems and residence patterns – probably influence the way bride price shapes women's status.

### 2.a Women as third-party agents in bride price

Women are not just ‘exchanged’ for bride price when they marry, they also have a stake in other people's marriages. First, it is often women who produce the valuables used in exchange, such as pigs, crops or mats. These can be used for other women's bride prices, allowing women to bring their female relatives into their husband's group, or to contribute to their son's marriage (Feil, 1981; Wardlow, 2002; Guo, 2020). Second, women play a role as relatives of the couple. In Lau, Malaita, in the Solomons, the groom's mother historically had a say in marriage arrangements because her daughter-in-law would assist her with subsistence and household work (Köngäs Maranda, 1974). On Ponam in Papua New Guinea, the bride's paternal kinswomen (such as her father's sisters) helped arrange marriages, trying to bring their brother's daughters into their husband's group (Carrier, 1993). Moreover, both parents may help with procuring valuables for a son's marriage (Demian, 2004). Finally, the mother of the bride often receives a share of the bride price, including the biggest and most valuable items (Goto, 1996; Dureau, 1998; Faugère, 2000, 2002; Maclean, 2010; Guo, 2014a, b, 2020; Paini & Gallo, 2018; Henry & Vávrová, 2020; Paini, 2020). In the Highlands, the mother of the bride receives the ‘mother's pig’ to thank her for raising her daughter (Strathern & Strathern, 1969; Wardlow, 2006b; Henry & Vávrová, 2016, 2020). In the Solomon Islands, the mother of the bride receives shell money to thank her for bearing, breastfeeding and raising her daughter (Goto, 1996; Dureau, 1998; Guo, 2014a, b, 2020). In the Loyalty Islands of New Caledonia, the groom's side presents an engagement gift to the bride and her parents to thank them for accepting the proposal (Faugère, 2000, 2002; Paini, 2020). At the wedding, the groom's family presents additional gifts to the bride's parents (Paini & Gallo, 2018; Paini, 2020). One of the terms for these gifts refers to the mother's breast milk (Paini, 2020). In Vanuatu, this is called *pem titi* (pay for breastfeeding).

### 2.b Relational power dynamics

Women occupy multiple social positions and their interests differ in relation to different ‘others’; they may start off relatively disempowered when they are young but become more empowered over time. Newlywed wives must prove themselves to their husbands and in-laws by working hard, assisting them and bearing and raising children (Köngäs Maranda, 1974; Pflanz-Cook, 1993; Salomon, 2000, 2002; Wardlow, 2006a; Jourdan & Labbé, 2020). In the early stages of the marriage, women are not yet fully accepted, have few rights, lack protection in domestic disputes and are at risk of mistreatment (Pflanz-Cook, 1993; Salomon, 2000). For example, Melpa husbands in Papua New Guinea could easily dismiss their wives for problems such as childlessness but repayment obligations were substantial if the woman left (Strathern & Strathern, 1969). However, as women meet expectations, bear children and show generosity and good conduct, their standing in the husband's family improves (Köngäs Maranda, 1974; Salomon, 2000, 2002; Jourdan & Labbé, 2020). Couples contribute to other people's marriages, children and exchange obligations and thereby grow their prestige (Zimmer-Tamakoshi, 1993). Motherhood also confers moral legitimacy and women can invoke it to make demands (Paini, 2003). Cultivating a successful marriage and bearing children eventually lowers or even nullifies repayment obligations (Strathern & Strathern, 1969; Henry & Vávrová, 2020; Jourdan & Labbé, 2020).

Having earned respect by contributing to subsistence and exchange, bearing children and managing family affairs, senior women contribute to the marriages of their sons, receive bride price for their daughters, and gain authority over their daughters-in-law. In the past, the mother-in-law monitored the wife's pregnancies and enforced compliance with modesty norms and domestic duties (Salomon, 2000, 2002; Salomon & Hamelin, 2008), reinforced by the fact that wives often lived with their female in-laws (Pflanz-Cook, 1993; Wardlow, 2006a). Senior women also gain more agency in exchange, and in some cases organise and contribute in their own right (Henry & Vávrová, 2016, 2020).

### 2.c Kinship structures

Bride price tends to be more widespread and more substantial in patrilineal and patrilocal societies (Goody & Tambiah, 1973; Anderson, 2007). Accordingly, it is often embedded in male-centric kinship structures that may reinforce its disadvantages. First, patriliny creates a patriarchal culture that disempowers women materially and ideologically. Patriliny also reinforces the bride price trap: as we have noted above (see Section 1.a), the fact that children belong to the father's group can prevent women from leaving (Köngäs Maranda, 1974; Spark, 2011). On Grande Terre in New Caledonia, fathers have traditionally had custody even when no bride price has been paid (Salomon, 2000). The fact that the wife hails from a different patriline can also fuel suspicion towards her, expressed in accusations of poisoning, sorcery or pollution (Strathern & Strathern, 1969; Salomon, 2000, 2002; see also Peacey et al., 2022). Moreover, some patrilineal societies enforce taboos that associate women with pollution, justifying their subordination and requiring them to observe gendered modesty norms (Köngäs Maranda, 1974; Burt, 1988; Salomon, 2000, 2002). While these beliefs have declined, women are still stigmatised for pregnancy complications and infertility (Salomon, 2000, 2002; Colleran, 2022). The idea that women are inferior also hurts them in disputes (Wardlow, 2006a).Second, patrilocal residence entails that women move away from their natal kin, which isolates them from their support networks, disadvantages them in disputes and makes them vulnerable to abuse (Köngäs Maranda, 1974; Zimmer-Tamakoshi, 1998; Spark, 2011). Among the Huli, patrilocal women also compete with the husband's relatives for his affection and resources, and the latter may bully the wife if they feel threatened (Wardlow, 2006a). In contrast, matrilineal and matrilocal women can draw on support from their own kin, which may explain why they are often less disadvantaged (see Chen et al., 2023; Reynolds et al., 2020 for example).

Some societies with matrilineal descent practise bride price, as is the case on Efate in Vanuatu and among the Maenge in New Britain (Panoff, 1978). However, others observe different marriage practices, suggesting that bride price effects cannot easily be disentangled from other aspects of the social system. In the Trobriand Islands in Papua New Guinea, residence is patrilocal but descent and land inheritance are matrilineal (Malinowski, 1929). Historically, people attributed conception not to the father but to matrilineal ancestor spirits, emphasised the mother's role in the continuation of the matriline and invoked the shared essence of matrilineal kin (Spiro, 1982; Brindley, 1984). Families exchange marriage gifts, but these do not constitute a bride price and relationships are not framed as men ‘owning’ women (Lepani, 2015). Men give annual harvest gifts to their married sisters and mothers (which also benefit their husbands); in this way, chiefs receive large gifts from their wives’ relatives (Leach, 1971; Malinowski, 1929). As the father's role in conception was denied (at an ideological level at least), these ‘groomprices’ are not a gender-swapped equivalent of bride price, which compensates the woman's kin for her reproductive capacities (Powell, 1969). Importantly, societies where bride price was historically less important and/or balanced out with equivalent return gifts could still be violent towards women, especially if they defied social norms (see Telefomin in Papua New Guinea; Jorgensen, 1993).

## 3. Changes in bride price dynamics

Contemporary marriage practices in Melanesia are not pristine traditions: they have been transformed by social changes underway since colonialism, such as conversion to Christianity, urbanisation, the expansion of formal education and the market economy (Marksbury, 1993). These have reshaped control over marriage, changing the way bride price impacts women's status. Again, its effects do not operate in a vacuum, but interact with various trends associated with ‘modernisation’ that may either reduce or amplify them.

### 3.a Changing levels of control over marriage

#### (De)regulation of reproduction

Many Melanesian societies have historically practised ‘pooled’ reproduction, in which kin groups harness the reproductive capacities of women to serve their shared interests (Colleran, 2022). Accordingly, they have often regulated sexuality to serve the interests of kin. Relatives imposed sanctions to protect the virginity of unmarried girls and restricted contact with the opposite sex (Burt, 1988; Rosi & Zimmer-Tamakoshi, 1993; Buchanan-Aruwafu et al., 2003; Wardlow, 2006a; Guo, 2006; Buchanan-Aruwafu & Maebiru, 2008; Zorn, 2010a; Jourdan & Labbé, 2020; Servy, 2020). Institutions such as men's houses reinforced gender segregation while belief in menstrual pollution discouraged sexual transgressions (Pflanz-Cook, 1993; Wardlow, 2006a; Wiessner & Pupu, 2021). Ritual seclusion, puberty initiations and bachelor's cults regulated young people's entry into the marriage market (Zimmer-Tamakoshi, 1993; Wiessner & Pupu, 2021). Relatives monitored young people and enforced prohibitions with fines, corporal punishment and social stigma (Rosi & Zimmer-Tamakoshi, 1993; Dureau, 1998; Buchanan-Aruwafu et al., 2003; Guo, 2006; Buchanan-Aruwafu & Maebiru, 2008).

Kin also arranged marriages (Strathern & Strathern, 1969; Köngäs Maranda, 1974; Carrier, 1993; Rosi & Zimmer-Tamakoshi, 1993; Zimmer-Tamakoshi, 1993; Jorgensen, 1993; Buchanan-Aruwafu et al., 2003; Wardlow, 2006a; Buchanan-Aruwafu & Maebiru, 2008; Salomon & Hamelin, 2008; Goddard, 2010; Zorn, 2010a; Henry & Vávrová, 2016; Eves, 2019; Guo, 2020; Paini, 2020; Sykes, 2020; Wiessner & Pupu, 2021). Partner choice was guided by preexisting kin ties, marriage alliances and the interests of kin groups, elders and community leaders (Carrier, 1993; Jorgensen, 1993; Marksbury, 1993; Pflanz-Cook, 1993; Wardlow, 2006a; Salomon & Hamelin, 2008; Wiessner & Pupu, 2021). Young people could also make advances, find partners at courtship parties, initiate engagements or veto matches proposed by their parents (Strathern & Strathern, 1969; Köngäs Maranda, 1974; Jorgensen, 1993; Pflanz-Cook, 1993; Wardlow, 2006a; Neuendorf, 2020). However, sometimes they were pressured (or forced) to abandon their wishes and accept a partner chosen for them (Jorgensen, 1993; Pflanz-Cook, 1993; Salomon, 2000; Salomon & Hamelin, 2008; Paini, 2020).

Missionaries initially opposed bride price, but most churches have come to accept it. As Christianity emphasises virginity before marriage, it now reinforces the way bride price legitimates female sexuality and modesty (Buchanan-Aruwafu et al., 2003; Wardlow, 2006a; Buchanan-Aruwafu & Maebiru, 2008; Zorn, 2010a; Guo, 2020; Jourdan & Labbé, 2020). However, in practice, women's reproductive careers are no longer as closely regulated by bride price. This shift may have been brought about by two distinct mechanisms.

First, many case studies suggest that ‘modernisation’ has undermined customary enforcement mechanisms. Colonial authorities opposed forced marriage and suppressed the harshest punishments such as feuding, kidnapping and homicide (Carrier, 1993; Jorgensen, 1993; Pflanz-Cook, 1993; Buchanan-Aruwafu et al., 2003). Missionaries also undermined cousin marriage and arranged marriage (Carrier, 1993). Missions and colonial administrations suppressed pollution beliefs and gender segregation, and promoted mixed-sex family homes, churches and schools (Wiessner & Pupu, 2021). Co-ed schooling and church activities, urban migration, mobile phones and motor transport have increased contact with the opposite sex (Carrier, 1993; Rosi & Zimmer-Tamakoshi, 1993; Buchanan-Aruwafu et al., 2003; Wardlow, 2006a; Buchanan-Aruwafu & Maebiru, 2008; Servy, 2013; Guo, 2020; Wiessner & Pupu, 2021). In diverse urban environments, youth also encounter people from other ethnolinguistic groups, which enlarges their dating pool (Marksbury, 1993; Rosi & Zimmer-Tamakoshi, 1993). These changes have weakened families’ control (Buchanan-Aruwafu et al., 2003; Buchanan-Aruwafu & Maebiru, 2008; Guo, 2020).

Second, globalisation may have spread liberal relationship models. Contact with foreign people and media has encouraged young couples to experiment (Buchanan-Aruwafu & Maebiru, 2008; Servy, 2013). Inspired by stories of romantic love and companionate marriage encountered in school, church and global mass media, many young people want to choose their own partners and enter love marriages (Rosi & Zimmer-Tamakoshi, 1993; Wardlow, 2006a; Servy, 2013).

#### Consequences of liberalisation

While relatives still try to influence partner choice, many young people now have premarital relationships or cohabit in *de facto* unions, which sometimes result in pregnancies (Carrier, 1993; Rosi & Zimmer-Tamakoshi, 1993; Buchanan-Aruwafu et al., 2003; Buchanan-Aruwafu & Maebiru, 2008; Goddard, 2010; Zorn, 2010a; Henry & Vávrová, 2016; Demian, 2017; Henry & Vávrová, 2020; Jourdan & Labbé, 2020; Paini, 2020; Servy, 2020; Wiessner & Pupu, 2021). While some marriages are still arranged, many young people enter love marriages based on romantic attraction, personal fulfilment and the partner's socio-economic status (Carrier, 1993; Jorgensen, 1993; Marksbury, 1993; Pflanz-Cook, 1993; Rosi & Zimmer-Tamakoshi, 1993; Biersack, 2010; Zorn, 2010a; Eves, 2019; Guo, 2020; Henry & Vávrová, 2016, 2020; Servy, 2013, 2020; Paini, 2020; Wiessner & Pupu, 2021). Many also marry partners without prior kin ties or from other islands and ethnolinguistic groups (Carrier, 1993; Jorgensen, 1993; Marksbury, 1993; Rosi & Zimmer-Tamakoshi, 1993; Zorn, 2010a; Biersack, 2010; Servy, 2020).

Elders often accept these decisions, for the sake of their grandchildren or to avoid conflict (Neuendorf, 2020; Servy, 2020; Wiessner & Pupu, 2021). Kin are more likely to accept a premarital pregnancy if the woman can support herself or she is in a relationship with a committed partner (Neuendorf, 2020). If parents do not approve of a match, couples sometimes deliberately become pregnant or elope to force their hand (Rosi & Zimmer-Tamakoshi, 1993; Salomon, 2002; Wardlow, 2006a; Servy, 2020). For many couples, bride price has become a *post hoc* affirmation rather than a precondition for embarking on their reproductive careers (Paini, 2020; Servy, 2020). While the written law does not protect cohabitation (Brown & Care, 2005), in practice it is increasingly treated as a valid alternative (Zorn, 2010a; Wiessner & Pupu, 2021). State courts sometimes award benefits to cohabiting partners and their children so as not to exclude them from vital resources such as land (Care & Brown, 2004). In sum, young people are taking control over their relationships, which may reduce the restrictive effects of bride price.

Some speculate that love matches could reduce domestic violence (Henry & Vávrová, 2020). However, these freedoms may also come at a cost. The legal rights of cohabiting couples remain uncertain, which can be destabilising, triggering litigation when a relationship breaks down or a spouse dies (Marksbury, 1993; Care & Brown, 2004). Moreover, cohabiting women may have a lower standing in the man's family. On Grande Terre, where trial marriages are customary, cohabiting girlfriends (for whom no bride price has been paid and who are not yet established in their partner's group) are most vulnerable to abuse (Salomon, 2000). As trial marriages become more common, so may these dynamics. Finally, the numbers of unstable relationships and premarital pregnancies have increased, along with single motherhood (Carrier, 1993; Servy, 2013; Widmer, 2013; Wiessner & Pupu, 2021). While married women are supported by their husbands, on Efate single mothers depend on their natal kin (Widmer, 2013; Brandl et al., 2023). Women who fall pregnant before they can care for a child or without a committed partner are stigmatised (Salomon, 2002; Widmer, 2013; Neuendorf, 2020).

### 3.b Changing levels of control over divorce

Under *kastom*, mothers may lose custody if they leave their husbands (see Section 1.a). Moreover, village courts headed by chiefs sometimes pressure women to remain with abusive partners to restore harmony (Biersack, 2016). However, *kastom* now coexists with the state, introduced under colonialism and expanded after independence, which can invalidate individual customs if they violate the constitution or the written law (Luluaki 1997; Care & Brown, 2004; Brown & Care, 2005; Jessep, 2010; Zorn, 2010a, b). As national constitutions affirm gender equality, government courts can overturn village court decisions that discriminate against women, although judges do not always address gender when ruling on such cases (Zorn, 2010b). Legal pluralism entails that couples can undergo a Christian, civil and/or a customary marriage ceremony, their rights defined accordingly by either *kastom* or statutory law (Care & Brown, 2004; Brown & Care, 2005; Zorn, 2010a). Formally registering a marriage grants women assets, maintenance and custody rights as they are defined for statutory marriages, which may exceed what they are owed under *kastom* (Care & Brown, 2004; Brown & Care, 2005).

These reforms may have loosened husbands’ and their families’ control over divorce, reducing the severity of the bride price trap. Women can now turn to state courts to obtain a divorce or gain custody (although statutory divorce is often fault-based and therefore not free from obstacles either, see Brown & Care, 2005). On Grande Terre, it was difficult for women to obtain a customary divorce without the agreement of both families and without losing their children (Salomon, 2000, 2002; Salomon & Hamelin, 2008). Nowadays, women can turn to French courts to register a statutory marriage, obtain a divorce and change a child's paternity and lineage affiliation to retain custody (Salomon, 2000, 2002). Moreover, courts often consider the ‘welfare principle’ (the best interest of the child) in custody disputes, which tends to favour the mother (Zorn, 2010a; Corrin, 2016; Jourdan & Labbé, 2020). Judges may combine *kastom* with the welfare principle to find a compromise, or they can overrule *kastom* to deprive a parent or issue no-contact orders if there is evidence of mistreatment (Brown & Care, 2005; Zorn, 2010a; Corrin, 2016). However, in many countries such reforms have triggered a backlash because they are perceived to disadvantage men in divorce, domestic violence and custody cases, introduce liberal values that go against *kastom* and Christianity, and destabilise families (Taylor, 2008; Gibbs, 2016; Biersack & Macintyre, 2016; Biersack, 2016).

### 3.c Changing levels of control over exchange

#### Individualisation of exchange

In many societies, grooms have traditionally assembled bride price with the help of their relatives, who produced valuables or acquired them from their exchange networks (Köngäs Maranda, 1974; Carrier, 1993; Zimmer-Tamakoshi, 1993; Akin, 1999; Faugère, 2002; Goddard, 2010; Macintyre, 2011; Servy, 2020; Jourdan & Labbé, 2020; Sykes, 2020; Henry & Vávrová, 2020). They thereby indebt themselves to relatives and senior men who control the circulation of valuables such as pigs or shell money (Carrier, 1993; Pflanz-Cook, 1993; Zimmer-Tamakoshi, 1993, 1998; Akin, 1999; Jourdan & Labbé, 2020). The couple then ‘owes’ these donors and must reciprocate by assisting them or sharing resources, and creditors may feel entitled to bully the wife (Köngäs Maranda, 1974; Carrier, 1993; Akin, 1999; Macintyre, 2011; Servy, 2020; Jourdan & Labbé, 2020). As debts are repaid, the woman's status increases (Zimmer-Tamakoshi, 1993, 1998).

Yet the market economy has provided young men with opportunities to build wealth through wage labour, cash cropping and business ventures (Carrier, 1993; Pflanz-Cook, 1993; Akin, 1999; Jourdan & Labbé, 2020). Successful men no longer need to indebt themselves to participate in gift exchange or assemble bride price (Carrier, 1993; Akin, 1999; Jourdan & Labbé, 2020). Some contribute more to their own bride price precisely to avoid indebting themselves to their relatives, or to protect their partners from entitlement dynamics (Macintyre, 2011; Jourdan & Labbé, 2020). The replacement of traditional valuables controlled by senior kin with cash has reinforced this trend (Carrier, 1993). Urban migration also contributes. Disconnected from their rural kin, urban men in Moresby do wage labour or odd jobs or borrow from friends to pay bride price (Goddard, 2010).

Individualised payments and control over decision-making can liken marriage more to a partnership between two individuals than an alliance between two families (Marksbury, 1993; Goddard, 2010; Jourdan & Labbé, 2020). While this may prevent women from indebting themselves to their in-laws, some have argued that it entrenches indebtedness to husbands. Wealthy Gende men in Papua New Guinea shoulder most of the payment alone (Zimmer-Tamakoshi, 1998). Feeling that the wife ‘owes’ him, the husband may disrespect and control her, especially if the payment was substantial (Zimmer-Tamakoshi, 1993, 1998). It is therefore unclear whether these changes really reduce entitlement dynamics, or whether they only shift their focus.

However, social change may grant other women greater agency over their own bride price. Tombema-Enga brides traditionally distribute their own bride price to relatives to thank them for supporting them, pay off debts and establish future exchange relationships (Feil, 1981). In the Solomons in contrast, historically only the bride's relatives played an active role in her bride price (Jourdan & Labbé, 2020). Today, some brides contribute to their own bride price or attempt to influence the amount, either to facilitate a specific match or to improve their standing with in-laws (Jourdan & Labbé, 2020). Educated urban women who can provide for themselves and are more independent are more likely to play an active role in their marriage arrangements (Marksbury, 1993; Jourdan & Labbé, 2020).

Young couples also play a greater role in the distribution of bride price. Often, bride price is distributed to the bride's parents, brothers, uncles and other relatives (Strathern & Strathern, 1969; Sykes, 2013, 2020; Jourdan & Labbé, 2020). In most places, the bride and groom traditionally do not receive a part of the bride price (Strathern & Strathern, 1969; Marksbury, 1993; Henry & Vávrová, 2020; Paini, 2020; but see Servy, 2020 for a counter-example). Nowadays, a share is sometimes given to the couple (Henry & Vávrová, 2016, 2020; Paini, 2020). This practice may have been started by missionaries, introducing Western gift-giving practices (Paini, 2020). Gifts emphasise the couple's status as an independent household (Henry & Vávrová, 2020; Paini, 2020), possibly reflecting neolocal residence in urban areas. Urban couples increasingly organise their own weddings where guests only give gifts to the couple (Paini, 2020). Kin obligations and associated gifts are still important in many places (Henry & Vávrová, 2020; Paini, 2020), but many young urbanites have weaker ties with their rural relatives, emphasise individual aspirations, concentrate resources in their nuclear families and form friendships with non-kin (Marksbury, 1993; Macintyre, 2011; Hukula, 2017; Jourdan & Labbé, 2020).

Young people may also gain more agency over return gifts. High-status families from Langalanga, Malaita, in the Solomons traditionally give an elaborate bridal gown made from shell money to the groom's parents, generating prestige for the bride's family and compensating in-laws should the bride displease them (Guo, 2020). Nowadays, the bride's kin may give the gown to the bride (Guo, 2020). She can sell it to support her household or fund her education and business ventures, improving her finances and possibly her bargaining power in the marriage (Guo, 2020).

#### Simplification of exchange

In many areas, *kastom* weddings have traditionally involved a complex series of transactions embedded in ongoing relationships between the two groups, including return gifts, gifts made for the couple's children, and further marriages between the two groups (Strathern & Strathern, 1969; Strathern, 1984; Pflanz-Cook, 1993; Maclean, 2010; Zorn, 2010a; Demian, 2004; Guo, 2020; Kelly-Hanku et al., 2016; Eves, 2019; Henry & Vávrová, 2016, 2020; Paini, 2020; Jourdan & Labbé, 2020). Some have argued that traditional bride price is therefore fundamentally about building ties between families (Macintyre, 2011; Eves, 2019). In the Highlands, large return gifts may also enhance the bride's status, protect her from mistreatment and make it easier to obtain a divorce (as only the balance needs to be repaid) (Strathern & Strathern, 1969; Henry & Vávrová, 2020). Brides with small return gifts may be more vulnerable (Henry & Vávrová, 2020).

In some settings, return gifts have declined and marriage exchanges have become one-way transactions with large, inflationary cash payments (Zorn, 2010a; Kelly-Hanku et al., 2016). Some researchers believe that this transforms bride price into a purchase, commodifying the woman and undermining her status in the marriage, and that this is the root cause of abusive entitlement dynamics, a sentiment shared by many locals (Marksbury, 1993; SPC, 2009; Zorn, 2010a; Macintyre, 2011; Kelly-Hanku et al., 2016; Eves, 2019). Within anthropology, debates about bride price have often focused on whether it is a gift or a commodity (see Evans-Pritchard, 1931; Dalton, 1966; Valeri, 1994; Jolly, 2015; see Box 3, SI). Others argue that cash gifts accomplish the same goals as traditional valuables and do not turn the exchange into a purchase (Faugère, 2000; Hess, 2009).

Alternatively, these changes may have been brought about by Christianity. On Simbo in the Solomons, brothers historically punished sisters for sexual transgressions but also supported them in disputes with their husbands and in-laws (Dureau, 1998). At marriage, the groom's and bride's parents engaged in a mutual exchange of gifts; husband and wife were equals who retained their natal lineage rights (Dureau, 1998). Colonial authorities and missions promoted nuclear families, undermined the basis of the brother–sister tie, and emphasised women's submission to their husbands (Dureau, 1998). Nowadays, the groom's parents ‘buy’ the bride in a one-way transfer of shell money and husbands have become more domineering (Dureau, 1998). Brothers are less supportive as their wives pressure them to prioritise them and their children, leaving their sisters stuck in troubled marriages (Dureau, 1998).

Both perspectives suggest that some aspects of ‘modernisation’ may increase entitlement dynamics, and that one-way exchanges are associated with devaluing women. Others imply the opposite. Marriage exchanges on Ponam used to involve a series of mutual gifts between the two families (Carrier, 1993). Over time, gifts from the bride's side declined as they were perceived to devalue the bride price – an insult to the bride and her kin (Carrier, 1993). Here, asymmetric exchanges were perceived to honour, not demean, the woman, suggesting that these dynamics may differ considerably depending on the local context.

### 3.d Changing levels of control over labour

The traditional division of labour creates interdependence between husbands and wives (see Section 2). Low-income Gende men still depend on their wives for subsistence labour and household management (Zimmer-Tamakoshi, 1998). As women are needed, they can easily find a new partner, but poor men's prospects for a second marriage are dim (Zimmer-Tamakoshi, 1998). Some have argued that men who depend on their wives are less likely to mistreat them (Zimmer-Tamakoshi, 1998; Wiessner & Pupu, 2021), improving women's bargaining power. Likewise, participating in the cash economy and earning their own money may improve women's economic agency (Marksbury, 1993). When Manga women became cash croppers, they became less dependent on their husbands’ incomes, empowering them to demand more help with childcare (Pflanz-Cook, 1993; but see Spark, 2011, 2014 on backlash against women who are perceived as too independent).

In contrast, where men dominate the market economy, they are less dependent on their wives (Zimmer-Tamakoshi, 1993, 1998; Wiessner & Pupu, 2021). Many men earn money through wage labour, business ventures and royalty payments from mining projects (Zimmer-Tamakoshi, 1993; Pflanz-Cook, 1993; Wardlow, 2006a). Money has become crucial for gift exchange, devaluing women's agricultural and handicraft work (Zimmer-Tamakoshi, 1993). Many women earn little, struggle to support themselves and depend on their husbands to meet their financial needs (Zimmer-Tamakoshi, 1993, 1998; Wardlow, 2006a; Goddard, 2010; Demian, 2017). This may encourage husbands to view their wives as dependents, undermining their status in the marriage and fuelling disrespect, exacerbated by the fact that wealthy men can easily find a new partner (Zimmer-Tamakoshi, 1993, 1998; Wiessner & Pupu, 2021). Women's lack of income also reinforces the bride price trap if they are unable to repay their bride price (see VWC, 2011). Finally, it may exacerbate tensions between in-laws if multiple women compete for the earnings of one man. Aspiring to companionate marriage (informed by mass media, Christianity and the desire for a ‘modern’ lifestyle), many Huli wives want their husbands to prioritise them and their children over their mothers and sisters (Wardlow, 2006a). As men have competing obligations, this can lead to tensions (Wardlow, 2006a).

## Conclusions

International bodies and non-governmental organisations maintain that bride price is a harmful cultural practice that reinforces gender-based violence and discrimination. Our review of the ethnographic record paints a more complex picture. Ethnographers have made a case that bride price has its downsides: it may restrict women's autonomy, fuel entitlement among husbands and in-laws, and trap women in troubled marriages. However, they have also highlighted potential benefits: it may secure women's access to resources and improve their social standing. Moreover, women are active players in bride price who are differently empowered in different relationships. ‘Bride price effects’ do not operate in a vacuum, and many other variables may influence outcomes related to it, including age, kinship structures and residence patterns. Modernisation reshapes already diverse bride price traditions in various ways, shifting how gifts are made, who makes and who receives them, who produces resources, and how relationships are formed in the first place. This transforms the power dynamics within families but may not move all levers in the same direction (or at least, different researchers have advanced conflicting ideas about what is happening). Bride price no longer regulates women's romantic relationships and reproductive careers as firmly as it did in the past. Market integration, urbanisation and formal education have enabled some young couples to gain more control over their own marriage, including the associated gift exchanges. However, whether forces associated with ‘modernisation’ (such as nuclearisation and the expansion of the cash economy) ultimately reduce or rather intensify disadvantages associated with bride price is somewhat unclear, and women's own success within the market economy may be an important factor in this equation.

In sum, the ethnographic record paints a rather ambiguous picture: the arguments are diverse and at times contradictory, defying attempts to draw a definitive conclusion that applies in all times and places. It is therefore unlikely that abolishing bride price will have unambiguously positive outcomes (see also Akurugu et al., 2022). Nevertheless, on account of its intensive engagement with communities, we can use the ethnographic record to identify potential mechanisms at play in sensitive issues such as bride price, along with predictions that can be tested with quantitative data.

For example, we can test the idea that high bride prices, especially when paid in cash, feed abusive entitlement dynamics. If bride price breeds a sense of ownership in husbands (see Section 1.a), then we expect women who are married with bride price to experience more domestic violence and coercive control than women without bride price. If larger amounts fuel *more* entitlement within a given society, then women with *larger* bride prices will suffer relatively *more* mistreatment. Moreover, if cash *commodifies* women (see Section 3.c), then women with larger cash payments might experience more mistreatment. In contrast, if high bride prices enhance women's standing (see Section 1.b), then women whose marriages involved *smaller* bride prices should experience *more* abuse. The relative importance of bride price can be tested against other predictors that we have identified, such as men's and women's participation in the market economy. If earning their own money empowers women (see Section 3.d), then women who do *not* earn money should be at *higher* risk of abuse.

**Figure 4.**
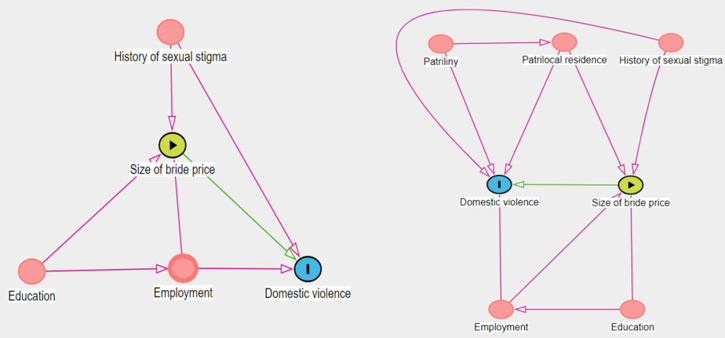
Left: Illustration of potential causal connections between women's education, employment, sexual stigma, bride price and domestic violence by way of a directed acyclic graph (yellow node = exposure variable, blue node = outcome variable, red node = variable that affects both exposure and outcome, green line = main effect to be analysed, purple line = open backdoor paths that can create spurious correlations between exposure and outcome, see Textor et al., 2016). This model implies that: to estimate the total effect of the size of bride price on domestic violence, the analysis has to adjust for employment and sexual stigma; a woman's sexual stigma is unrelated to her education and employment status; and after adjusting for employment, bride price and sexual stigma, education is unrelated to domestic violence. Right: Illustration of potential causal connections between women's education, employment, sexual stigma, residence, kinship, bride price and domestic violence. This model implies that: to estimate the total effect of the size of bride price on domestic violence, the analysis has to adjust for employment, sexual stigma and residence; a woman's history of sexual stigma is unrelated to her education, employment, kinship system and residence pattern; a woman's education and employment are unrelated to her residence and kinship pattern; after adjusting for employment, bride price, sexual stigma and residence, education is unrelated to domestic violence; and after adjusting for residence, kinship pattern is unrelated to bride price. Note that in either case, these assumptions may or may not reflect reality; this is merely an example of some of the predictions that *could* be tested.

These are just some of the many concrete hypotheses that could be derived from the ethnographic record. Importantly, marriage practices vary across societies, which must be considered when formulating hypotheses for a particular setting. Quantitative researchers can then test these predictions by collecting systematic data from women about how their bride price was paid, how large it was, and about their experiences within the marriage, along with socio-economic factors such as their source of income. Causal inference can be made more robust (and erroneous conclusions based on spurious correlations avoided) by formalising potential relationships between different predictors, which interact with each other and may therefore confound results. We can visualise these relationships with the help of directed acyclic graphs (DAGs). Directed acyclic graphs are causal diagrams composed of nodes (denoting variables) connected by directed edges (causal arrows leading from one node to another) without cycles (i.e. paths starting from a node do not lead back to it) (Pearl et al., 2016). Unlike informal flowcharts (which may encode anything from concrete causal relationships to broad theoretical concepts and non-causal connections), DAGs formalise assumptions about the underlying causal structure of a phenomenon to guide data analysis (Pearl et al., 2016). For example, as illustrated in the left-hand DAG in Figure 4, educated women may attract higher bride prices while their job opportunities make them less financially dependent on their husbands, protecting them from mistreatment. Alternatively, women with a history of sexual stigma may fetch lower bride prices and are subsequently mistreated, but causally, the stigma itself may be as important as or even more important than bride price. Associations between bride price and violence are thus not necessarily causal and may be driven by underlying factors that shape both. To statistically estimate the size of any causal effect of bride price, we therefore need to know what other variables to adjust for based on these interdependencies. Note that this is a single instance of a causal model, and not all variables can or should be in one model. Individual models can be tested against alternatives that make different assumptions about the mechanisms that drive an outcome and how they are connected to each other in a specific sample. For example, the above model can be tested against a more complex one (such as the right-hand DAG in Figure 4) in which the risk of domestic violence also increases with patrilocal residence and patriliny (see Section 2.c).

Taking ethnographic *arguments* seriously allows us to identify relevant claims in the literature and to challenge reductive narratives. These arguments can inform quantitative research by generating novel hypotheses, drawing attention to competing predictions, raising awareness of potential confounders that may have gone unnoticed in other disciplines and encouraging critical reflection about causality. Rather than parachuting into the field with assumptions based on decontextualised models, we can use verbal models advanced by ethnographers to refine quantitative study designs. As the social sciences become more aware of challenges posed by causal inference, researchers increasingly incorporate explicit causal models into their workflow. Here, we make a case for integrating ethnography into that process.

## Supporting information

Brandl and Colleran supplementary material Brandl and Colleran supplementary material

## Data Availability

n/a.

## Supplementary material

To view supplementary material for this article, please visit https://doi.org/10.1017/ehs.2024.21

## Research transparency and reproducibility

n/a.
