# Supplementary material for: Does bride price harm women? Using ethnography to think about causality
Source: Evol Hum Sci. 2024 May 3;6:e29. doi: 10.1017/ehs.2024.21 (PMC11363001; doi:10.1017/ehs.2024.21)
Supplement: Brandl and Colleran supplementary material [file S2513843X24000215sup001.docx]

**Supplementary Information.**

Table of Contents

**1. Field Sites**2

**2. Supplementary Boxes**3

**3. Summary Tables**5

**4. References**7

**1. Field Sites**

| **Country** | **Region or Area** | **Society or Location** | **References** |
| --- | --- | --- | --- |
| Papua New Guinea | Highlands  Enga  Western  Jiwaka  Eastern  Hela  Southern | Enga  Mt Hagen  Melpa  Manga  Anglimp-South Waghi  Maring  Unggai-Bena  Goroka  Huli  Mendi  Wiru  Kewa | Wiessner & Pupu, 2021; Merlan, 1988; Feil, 1981  Henry & Vávrová, 2020; 2016; Stewart & Strathern, 2005; 1998; Merlan, 1988; Wilson, 1987  Strathern & Strathern, 1969  Pflanz-Cook, 1993  Eves, 2019  Maclean, 2010  Kelly-Hanku *et al.*, 2016  Spark, 2014, 2011 (?); Macintyre, 2011; Zimmer-Tamakoshi, 1998  Wardlow, 2006a; 2006b; 2002  Merlan, 1988  Clark, 1991  Josephides, 1985; 1983 |
|  | South  Western  Gulf  Central  Milne Bay | Kiunga  Baimuru  Port Moresby  Suau  Trobriand Islands | Gibbs, 2016  Neuendorf, 2020  Demian, 2017; Hukula, 2017; Spark, 2014; 2011 (?); Macintyre, 2011; Goddard, 2010; Rosi & Zimmer-Tamakoshi, 1993  Demian, 2004  Lepani, 2015; Powell, 1969; Malinowski, 1929 |
|  | Momase  Madang  Morobe  Sandaun | Madang  Gende  Lae  Telefomin | Macintyre, 2011  Zimmer-Tamakoshi, 1993  Macintyre, 2011  Jorgensen, 1993 |
|  | Islands | Lihir  Kavieng (New Ireland)  New Britain  Ponam | Macintyre, 2011  Macintyre, 2011  Panoff, 1978  Carrier, 1993 |
| Solomon Islands | Guadalcanal | Honiara | Jourdan & Labbé, 2020; Guo, 2020; 2014a; 2014b; Buchanan-Aruwafu *et al.*, 2003 |
|  | Malaita | Langalanga Lagoon  Auki  Lau Lagoon  Kwara’ae  Kwaio | Guo, 2020; 2014a; 2014b; 2006; Goto, 1996  Buchanan-Aruwafu & Maebiru, 2008; Buchanan-Aruwafu *et al.*, 2003  Köngäs Maranda, 1974  Burt, 1988  Akin, 1999 |
|  | New Georgia Islands | Simbo | Dureau, 1998 |
| Vanuatu | Efate | Port Vila  Pango | Servy, 2020; 2013; Widmer, 2013  Widmer, 2013 |
|  | Espiritu Santo | Luganville | Taylor, 2008 |
|  | Vanua Lava | Vêtuboso | Hess, 2009 |
|  | Malakula | Big Nambas | Colleran, 2022 |
| New Caledonia | Loyalty Islands | Lifou  Maré | Paini, 2020; 2003; Paini & Gallo, 2018  Faugère, 2002; 2000 |
|  | Grande Terre | A‘jië, Paicî | Salomon & Hamelin, 2008 (?); Salomon, 2002; 2000 |
| West Papua | Central Papua | Wodani | Breton, 1999 |
| Australia | Queensland | Cairns | Sykes, 2020; 2018; 2013 |

**Supplementary Table S1.** Field sites covered in this review. This table only includes publications based on field work, not reviews or works based on archival records. Full references are provided in the main text. (?) indicates that field sites are not explicitly stated but were inferred from the author’s publication record.

**2. Supplementary Boxes**

**Box 2. Quantitative Evidence**

**Africa and Asia:** In Ghana, communities disapprove of women who use contraception without informing their husbands, especially when the bride price has been paid in full (Horne *et al.*, 2013). The woman’s family will also be less likely to object if her husband responds with violence (Horne *et al.*, 2013). But in the Democratic Republic of Congo, high bride prices are associated with *more* marital satisfaction and *less* support for domestic violence among women (Nunn & Lowes, 2018). In Zambia and Indonesia, bride price is associated with higher levels of parental investment in the education of daughters (Ashraf *et al.*, 2020). The relevant literature is more expansive than the works cited here; these are intended as a snapshot of contradictory findings, not an exhaustive account of the topic.

**Melanesia:** Solomon Islands women who have married with bride price are more likely to experience domestic violence than women without bride price, but women whose bride price has been paid in part, not in full, are most at risk (SPC, 2009). In Vanuatu by contrast, intimate partner violence is not statistically associated with women’s marital status or whether their bride price has been paid in full (VWC, 2011). While about 53% of ni-Vanuatu women agree with the statement that the wife becomes the husband’s property once bride price has been paid, only 32% feel that this justifies domestic violence (VWC, 2011). 78% agree that wives have a right to refuse intercourse; while mentioning bride price lowers agreement, 60% hold firm on this position even if bride price is paid (VWC, 2011). 84% believe that bride price improves the way they are treated by husbands and in-laws (VWC, 2011). Opposition to gender-based violence appears to be strongest in New Caledonia: 93% of Kanak women disagree with the idea that husbands have a right to commit domestic violence, and 96% state that refusing intercourse is not a legitimate reason for violence (Salomon & Hamelin, 2008).

**Box 3. Gifts or Commodities?**

It is sometimes implied that if bride price is a commodity (like money), it must have a commercial motive and therefore harms women. Conversely, if it is a gift (given without a profit motive), for example traditional valuables like pigs, it must be about establishing ties, and therefore empowers women. Most development professionals treat bride price as a commodity because a woman is ‘exchanged’ for an object (Jolly, 2015). In contrast, most anthropologists treat bride price as a gift, insist that it is fundamentally different from commercial transactions, and reject the view that it treats the bride like a commodity that can be bought and sold (Evans-Pritchard, 1931; Dalton, 1966; Jolly, 2015). This argument is based on its embeddedness in reciprocal exchanges and ongoing relationships between the two families, along with the fact that marriage ties the woman to one husband and imbues her with value (Evans-Pritchard, 1931; Dalton, 1966; Jolly, 2015). On these grounds, some anthropologists have rejected the term ‘bride price’ in favour of ‘bride wealth’ (Evans-Pritchard, 1931; Dalton, 1966). Both perspectives construct a dichotomy between gifts and commodities, with the assumption that gift exchanges give women value and agency whereas commodity exchanges devalue them (Jolly, 2015). Other researchers have challenged this dichotomy (Valeri, 1994; Jolly, 2015). The premise of the debate is problematic: it presumes that bride price has a ‘true’ nature as either a gift or a commodity, that this nature creates inherent social consequences, and that we have to excavate this nature to uncover these consequences. This perspective essentializes and reifies culture, makes ahistorical, exoticizing assumptions, and entrenches false dichotomies between ‘local’ and ‘foreign’ or ‘authentic’ and ‘inauthentic’ practices (Jolly, 1992). These tensions are also evident among women’s rights campaigners, some of whom blame gender-based violence on patriarchal customs while others blame colonialism (which allegedly undermined egalitarian institutions) (Salomon & Hamelin, 2008; Taylor, 2008). These narratives can lead to futile debates where adverse outcomes are either blamed on ‘patriarchal tradition’ (i.e. insufficient modernization) or ‘capitalist modernity’ (i.e. the abandonment of tradition). The former infuses the missionaries’ antipathy towards ‘backwards’ customs with a humanitarian tinge, whereas the latter filters cultural conservatism through an anti-capitalist lens. Within anthropology, both are best treated not as analytical tools but as master narratives about social change in post-colonial countries, where either ‘savage’ cultures must be civilized by modernization, or ‘noble savages’ were corrupted by colonialism. Both perspectives attribute harm to vague constructs like ‘tradition’ or ‘modernity’, leaving little room for nuance. Instead, researchers should ask what people use bride price for and how conflicting interests motivate them to contest its implications. On Vanua Lava in Vanuatu, wedding guests assert that bride price does not entail the purchase of a woman, yet husbands sometimes invoke the fact that they have ‘paid’ to assert dominance (Hess, 2009). Accordingly, people from the same society flexibly use different interpretations of bride price in different circumstances to accomplish their goals.

**3. Summary Tables**

| **Core argument about bride price** | **Mechanism** | **Gendered consequences** |
| --- | --- | --- |
| Restricts legitimate female sexuality to marriage. Premarital transgressions damage a girl’s reputation. | Reputation | Bride’s kin restrict her premarital conduct. |
| Brings wealth to bride’s family. Premarital transgressions damage a girl’s bride price. | Family enrichment |  |
| Builds ties between families that benefit the bride’s family. | Advantageous family ties | Bride’s kin exercise control over partner choice. |
| Obligates wife to perform subsistence and domestic labour and bear children. | Transfer of rights over fertility and labour | Male and affinal entitlement. Male reproductive control |
| Repayment in cases of divorce. | Economic burden on women | Trapping effect. Wives with high bride prices are more likely to be trapped. |
| Ties between families are socially significant and benefit bride’s family. | Advantageous family ties |  |
| Secures children’s patrilineage affiliation. Husband’s family has custody rights. | Custody rights |  |
| Grooms are under financial pressure to meet the demands of the bride’s family, recruit and reciprocate donors, shoulder payment, or compete with rivals. | Economic burden on men | Men either fail to realize a desired match or are burdened financially if they do. Stressed men abuse women before and during the marriage. |
| Values women’s contributions to production, reproduction, and alliance formation. High prices raise wife’s standing with husband’s kin. | Social standing and respect | Bride’s social status increases once married. Wives with high bride prices less likely to experience abuse. |
| Secures woman’s right to investment from husband’s kin, including access to resources, land. | Usufruct and support rights | Safety net. |
| Secures children’s patrilineage affiliation; rights to maintenance, protection, inheritance, i.e. investment from fathers and paternal kin. | Maintenance and inheritance rights |  |
| Repayment in cases of divorce. | Economic burden on women | Divorce is costly for both sexes, which stabilizes marriages and secures the safety net. |
| Husbands and their kin must pay for a second bride price if they want to remarry. | Economic burden on men |  |
| **Core argument about other variables** |  |  |
| Different lineage membership fuels suspicion towards the wife. | Patrilineal kinship structures | Female subordination. |
| Children belong to the father’s patriline. |  | Trapping effect. |
| Social norms, along with ideologies of female pollution and inferiority, reinforce male domination. | Patriarchal social norms | Female subordination. |
| Isolates wives from natal kin, support networks. | Patrilocal residence |  |
| Creates competition with co-resident in-laws. |  |  |

**Supplementary Table S2.** Effects of bride price and other variables on women’s status. Definitions: *Male and affinal entitlement*: Husbands and in-laws feel entitled to control the wife’s labour and fertility and abuse her if their expectations are not met. *Trapping effect*: Women are trapped in their marriages because their natal kin do not support the divorce or because they do not want to leave children behind. *Male reproductive control*: Fertility reflects interests of the husband and/or his family. *Female subordination*: Women are subordinated to men, disadvantaged in disputes, blamed for pregnancy-related misfortunes, and/or vulnerable to abuse from husbands and in-laws. *Safety net*: Married women and their children enjoy more social and material security.

| **Core argument** | **Mechanism** | **Gendered consequences** |
| --- | --- | --- |
| Customary enforcement mechanisms for sexual relationships (punishments, gender segregation) deteriorate under social change beginning with colonialism. | State formation, Christianization, education, migration and urbanization, new communication and transport technologies | Individualization of marriage. Premarital relationships, cohabitation, and love marriage increase, but this may compromise the safety net. |
| Cultural transmission of liberal Western relationship models through globalization. | Foreign media and people, Christianization, education |  |
| Colonial introduction of a formal legal system managed by the state favours women in custody cases. | State formation | Reduces trapping effect but may destabilize relationships. |
| Market economy increases financial autonomy of successful men, making them less dependent on kin for bride price. | Market integration | Individualization of marriage. Wives less indebted to in-laws but more to husbands, concentrating female subordination (if it continues) in husbands. |
| Urban migration removes men from rural kin networks, forcing them to pay their own bride prices and into alternative arrangements for payment. | Migration and urbanization | Individualization of marriage. Increase in legally ambiguous and unstable relationships. |
| Growing education and job prospects for women increase their autonomy vis-à-vis kin. | Market integration and education | Individualization of marriage. Brides play more active role in bride price. |
| More neolocal residence, esp. in urban areas. Possibly within the context of less kin-dense social networks. | Migration and urbanization | Bride and groom increasingly likely to receive a part of the bride price and/or return gift, increasing their financial autonomy. Individualization of marriage. |
| Cultural transmission of Western gift-giving practices through missionaries. | Christianization |  |
| Simplification of exchange due to greater access to money. | Market integration | Wives commodified, promoting female subordination. |
| Shift of control from natal kin to husbands and in-laws due to Christian emphasis on the nuclear family. | Christianization | Marriage less egalitarian, promoting female subordination. Simplification of exchange. |
| Market economy increases financial autonomy of successful men vis-à-vis women. | Market integration | Wives depend on husbands, promoting female subordination. Reinforces trapping effect. |
|  |  | Wives compete with female in-laws, promoting female subordination. |
| Market economy increases financial autonomy of successful women vis-à-vis men. | Market integration and education | Wives empowered in the marriage, enabling them to extract more commitments from their husbands. |

**Supplementary Table S3.** Effects of modernization on bride price dynamics. Definitions: *Individualization of marriage*: Role of relatives and their control over marriage declines, making marriage more like a partnership between two individuals than like an alliance between two families. *Simplification of exchange*: Shift from mutual exchange of traditional valuables to one-way transfer of cash.

**4. References**

Ashraf, N., Bau, N., Nunn, N., & Voena, A. (2020). Bride Price and Female Education. *The Journal of Political Economy*, *128*(2), 591–641. <https://doi.org/10.1086/704572>

Evans-Pritchard, E. E. (1931). 42. An Alternative Term for “Bride-Price.” *Man*, *31*, 36–39. <https://doi.org/10.2307/2789533>

Dalton, G. (1966). “Bridewealth” vs. “Brideprice.” *American Anthropologist*, *68*(3), 732–738. <https://doi.org/10.1525/aa.1966.68.3.02a00070>

Hess, S. (2009). *Person and place: ideas, ideals and the practice of sociality on vanua lava, vanuatu (person, space and memory in the contemporary pacific)* (1st ed., Vol. 2, pp. xiv–xiv). Berghahn Books. <https://doi.org/10.1515/9781845459390>

Horne, C. Dodoo, F. N.-A., & Dodoo, N. D. (2013). The Shadow of Indebtedness: Bridewealth and Norms Constraining Female Reproductive Autonomy. *American Sociological Review*, *78*(3), 503–520. <https://doi.org/10.1177/0003122413484923>

Jolly, M. (1992). Specters of Inauthenticity. *The Contemporary Pacific*, *4*(1), 49–72.

Jolly, M. (2015). Braed Praes in Vanuatu: Both Gifts and Commodities?: Braed Praes in Vanuatu. *Oceania*, *85*(1), 63–78. <https://doi.org/10.1002/ocea.5074>

Nunn, N., & Lowes, S. (2018). Bride Price and the Well-Being of Women. In *Towards Gender Equity in Development*. Oxford University Press. <https://doi.org/10.1093/oso/9780198829591.003.0006>

Salomon, C., & Hamelin, C. (2008). Challenging Violence: Kanak Women Renegotiating Gender Relations in New Caledonia. *The Asia Pacific Journal of Anthropology*, *9*(1), 29–46. <https://doi.org/10.1080/14442210701822191>

Secretariat of the Pacific Community (SPC) (2009). *Solomon Islands Family Health and Safety Study: A Study on Violence against Women and Children*. Noumea, New Caledonia: Secretariat of the Pacific Community.

Taylor, J. P. (2008). The Social Life of Rights: “Gender Antagonism”, Modernity and Raet in Vanuatu. *The Australian Journal of Anthropology*, *19*(2), 165–178. <https://doi.org/10.1111/j.1835-9310.2008.tb00120.x>

Valeri, V. (1994). Buying Women But Not Selling Them: Gift and Commodity Exchange in Huaulu Alliance. *Man (London)*, *29*(1), 1–26. <https://doi.org/10.2307/2803508>

Vanuatu Women's Centre (VWC) (2011). *Vanuatu National Survey on Women’s Lives and Family Relationships*. Port-Vila, Vanuatu. <https://pacificwomen.org/research/vanuatu-womens-centre-overview-on-violence-against-women/>
